# Six new species and a new record of *Linan* Hlaváč in China, with a key to species (Coleoptera, Staphylinidae, Pselaphinae)

**DOI:** 10.3897/zookeys.793.27661

**Published:** 2018-10-29

**Authors:** Yu-Qing Zhang, Li-Zhen Li, Zi-Wei Yin

**Affiliations:** 1 Department of Biology, Shanghai Normal University, 100 Guilin Road, Shanghai, 200234, P. R. China Shanghai Normal University Shanghai China

**Keywords:** China, distribution, identification key, *
Linan
*, new record, new species, Tyrini

## Abstract

Six new species of the genus *Linan* Hlaváč are described from central to southern China: *L.arcitibialis***sp. n.** (Hubei), *L.denticulatus***sp. n.** (Guizhou), *L.divaricatus***sp. n.** (Jiangxi), *L.geneolatus***sp. n.** (Guizhou), *L.mangshanus***sp. n.** (Hunan), and *L.mulunensis***sp. n.** (Guangxi), with illustrations of habitus and major diagnostic characters. *Linanmegalobus* Yin & Li, originally described from Guizhou, is newly recorded in Hubei. An updated key to and a distributional map of all 16 known species are provided.

## Introduction

The pselaphine genus *Linan* Hlaváč belongs to the tribe Tyrini that is comprised of ten species predominantly distributed in China (with one species extending southwards to northern Thailand; Figure 11) (Hlaváč 2003; Yin et al. 2011, 2013; Yin and Li 2012, 2013). Members are characterized by the head lacking or with indistinct vertexal and frontal foveae, laterally expanded or protuberant maxillary palpomeres II–IV, roughly punctate head and pronotum, lack of a transverse antebasal sulcus on the pronotum, and presence of a median metaventral fovea. Based on presence or absence of modification on male antennomeres IX–X, two species groups were defined (Yin and Li 2013). The current diversity of *Linan* remains underexplored. Here we report the discovery of six new species based on the material from various areas in China collected after 2012. The species number of *Linan* now rises to 16, we accordingly provide an updated identification key and distributional maps for the genus.

## Material and methods

All material treated in this study is housed in the Insect Collection of Shanghai Normal University, Shanghai, China (**SNUC**).

The label data of the material are quoted verbatim, additional information is included in parentheses. Dissected parts were preserved in Euparal on plastic slides that were placed on the same pin with the specimen. The habitus image was taken using a Canon 5D Mark III camera in conjunction with a Canon MP-E 65mm f/2.8 1–5× Macro Lens, and a Canon MT-24EX Macro Twin Lite Flash was used as light source. Images of the morphological details were produced using a Canon G9 camera mounted to an Olympus CX31 microscope under transmitted light. Zerene Stacker (version 1.04) was used for image stacking. The base map was produced from http://www.simplemappr.net/ (Shorthouse 2010). All images were optimized and grouped into plates in Adobe Photoshop CS5 Extended.

The following acronyms are used in the text: BL–length of the body (= HL + PL + EL + AL); HL–length of the head from the anterior clypeal margin to the occipital constriction; HW–width of the head across eyes; PL–length of the pronotum along the midline; PW–maximum width of the pronotum; EL–length of the elytra along the suture; EW–maximum width of the elytra; AL–length of the dorsally visible part of abdomen along the midline; AW–maximum width of the abdomen. Paired structures are treated as singular, except for eyes, metaventral processes, and parameres which are treated as plural.

## Taxonomy

### Linan arcitibialis sp. n.

Taxon classificationAnimaliaColeopteraStaphylinidae

http://zoobank.org/2C08CE31-68A0-4BEE-BAF3-BD9B3A1B6009

Figs 1A , 2 , 11A

#### Type material.

(36 ♂♂, 40 ♀♀). **Holotype: CHINA**: ♂: ‘China: Hubei, Enshi Tujia and Miao Autonomous Prefecture, Xingdoushan N. R. (星斗山自然保护区), San-xian-chang (三县场), 30°2'20.48"N, 109°8'33.89"E, 1114 m, 20.v.2017, sift, Zhou GC, Tian T, & Huang ZG leg.’ (SNUC). **Paratype: CHINA**: 4 ♂♂, 8 ♀♀, same label data as holotype; 15 ♂♂, 16 ♀♀, same label data, except ‘19.v.2017’; 11 ♂♂, 15 ♀♀, same label data, except ‘30°2'46.03"N, 109°7'49.39"E, 1205 m, 18.v.2017’; 1 ♂, same label data, except ‘30°2'29.98"N, 109°8'1.60"E, 1253 m, 18.v.2017’; 1 ♂, ‘China: Hubei, Enshi Tujia and Miao Autonomous Prefecture, Changtanhe (长潭河), Lianghekou Village (两河口村), 30°0'6.00"N, 109°44'27.36"E, 1234 m, 14.v.2017, sift, Zhou GC, Tian T, & Huang ZG leg.’; 1 ♂, ‘China: Hubei, Enshi Tujia and Miao Autonomous Prefecture, Xianfeng Hsien (咸丰县), Huangjindong Country (黄金洞乡), Maliuxi Village (麻柳溪村), 29°57'34.15"N, 109°1'15.82"E, 752 m, 24.viii.2017, sift, Zhou GC & Irfan M leg.’; 2 ♂♂, 1 ♀, ‘China: Chongqing City, Pengshui Hsien(彭水县), Moweishan Scenic Spot (摩围山风景区), 29°11'11.15"N, 108°2'59.32"E, 1234 m, 1568 m, 25.vii.2017, sift, Zhou GC & Irfan M leg.’ (all in SNUC).

#### Diagnosis of male.

Length 2.71–2.77 mm; antennomere IX expanded laterally, with small process near apex; long metaventral processes narrowing toward apex; protibia with distinct apical spine; mesotibia strongly arched; metatrochanter with short, blunt ventral projection.

#### Description.

**Figure 1.**
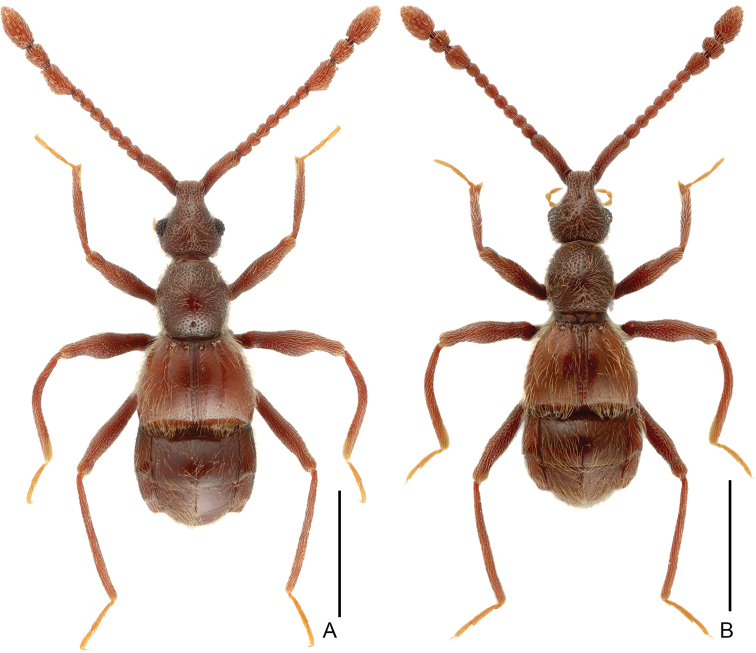
Dorsal habitus of *Linan* species. **A***L.arcitibialis* sp. n. **B***L.denticulatus* sp. n. Scale bars: 1 mm.

**Figure 2.**
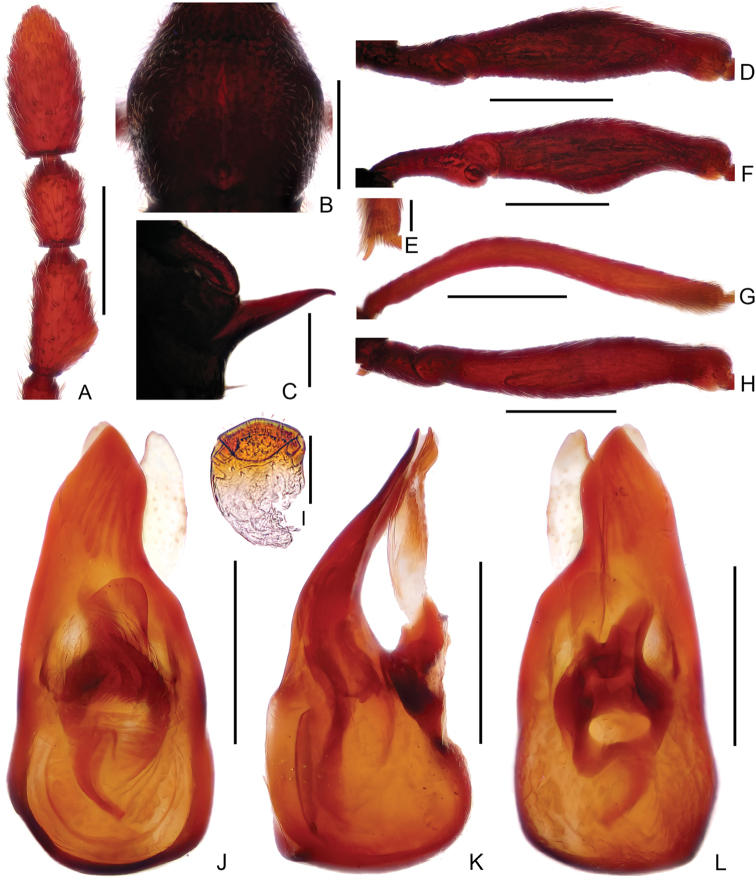
Diagnostic features of male *Linanarcitibialis* sp. n. **A** Antennal club **B** Pronotum **C** Metaventral process, in lateral view **D** Protrochanter and profemur **E** Apex of protibia **F** Mesotrochanter and mesofemur **G** Mesotibia **H** Metatrochanter and metafemur **I** Sternite IX **J–L** Aedeagus, in dorsal view (**J**), lateral (**K**), and ventral (**L**) view. Scale bars: 0.3 mm (**A, B, D, F, G, H**); 0.2 mm (**C, J, K, L**); 0.05 mm (**E**); 0.1 mm (**I**).

Male (Figure 1A). Length 2.71–2.77 mm. Head slightly longer than wide, HL 0.62–0.63 mm, HW 0.55–0.56 mm; eyes small, each composed of about 24 facets. Antennal scape elongate, about 3.3 times as long as wide, antennomeres II–VIII similar, each about as long as wide, antennomere IX (2A) much longer than wide, angularly expanded laterally at basal third, with small rounded process near apex, antennomeres X–XI simple. Pronotum (Figure 2B) about as long as wide, PL 0.57–0.58 mm, PW 0.55–0.57 mm. Elytra much wider than long, EL 0.60–0.63 mm, EW 0.93–0.95 mm. Metaventral processes (Figure 2C) long, narrowing apically. Protrochanter and profemur simple (Figure 2D), protibia with small but distinct spine (Figure 2E) at apex; mesotrochanter simple, mesofemur expanded ventrally at middle (Figure 2F), mesotibia (Figure 2G) strongly arched at basal 2/5; metatrochanter (Figure 2H) with short, blunt ventral projection. Abdomen slightly wider than elytra, AL 0.92–0.93 mm, AW 0.95–0.98 mm; tergite IV about twice as long as tergite V; sternite IX as in Figure 2I. Length of aedeagus (Figure 2J–L) 0.49 mm; median lobe asymmetric, narrowing apically; elongate parameres moderately broadened dorso-ventrally, narrowed at apex.

Female. Similar to male in general morphology; eyes each composed of about 24 facets; antennae and legs simple; lacking metaventral processes. Measurements: BL 2.47–2.69 mm, HL 0.57–0.60 mm, HW 0.50–0.51 mm, PL 0.53–0.55 mm, PW 0.52–0.55 mm, EL 0.48–0.59 mm, EW 0.92–0.99 mm, AL 0.89–0.95 mm, AW 0.95–0.99 mm.

#### Distribution.

China: Hubei (Figure 11A).

#### Etymology.

The new specific epithet refers to the strongly arched mesotibiae.

#### Comparative notes.

The new species is placed as a member of the *L.cardialis*-group based on the modified male antennomere IX. *Linanarcitibialis* is the only member of the group that exhibits a simple antennomere X. Combined with the unique form of antennomere IX and strongly arched mesotibia, males of this species can be readily separated from all other congeners at a quick glance.

### Linan denticulatus sp. n.

Taxon classificationAnimaliaColeopteraStaphylinidae

http://zoobank.org/CC051B42-2913-471B-8F88-FFE9E6963CD8

Figs 1B , 3 , 11B

#### Type material.

(1 ♂, 1 ♀). **Holotype: CHINA**: ♂: ‘China: N. Guizhou, Daozhen County (道真县), Dashahe N. R. (大沙河自然保护区), 29°10'12"N, 107°33'36"E, mixed leaf litter, sifted, 1730 m, 07.VII.2015, Jiang, Peng, Tu, & Zhou leg.’(SNUC). **Paratype: CHINA**: 1 ♀, same label data as the holotype, (SNUC).

#### Diagnosis of male.

Length 2.61mm; antennomeres IX–XI enlarged, lacking obvious modification; short metaventral processes narrowing toward apex, area above metacoxae projecting; protibia with distinct apical spine; metatrochanter with blunt, apically curved ventral projection.

#### Description.

**Figure 3.**
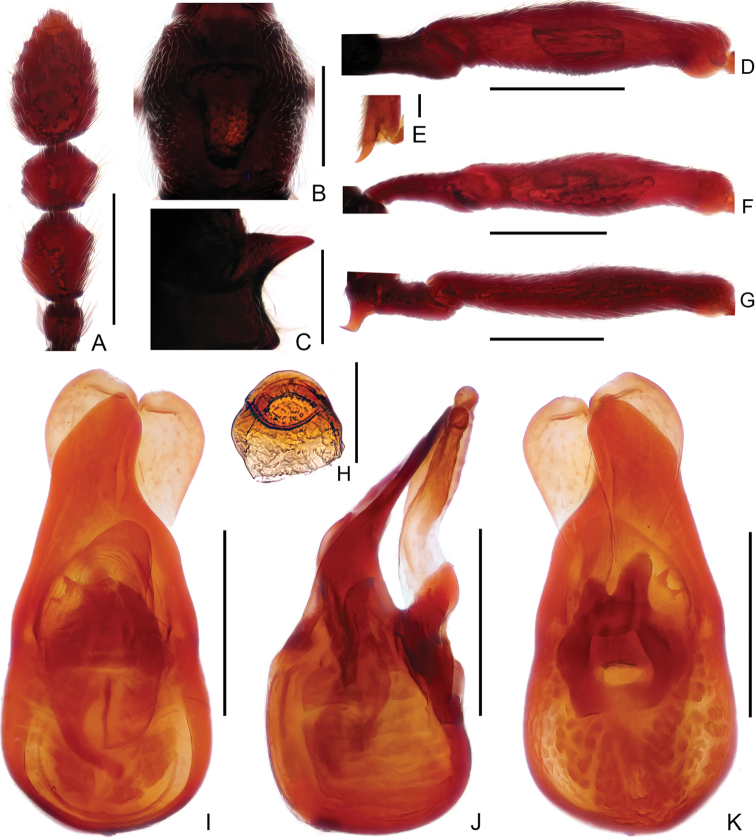
Diagnostic features of male *Linandenticulatus* sp. n. **A** Antennal club **B** Pronotum **C** Metaventral process, lateral view **D** Protrochanter and profemur **E** Apex of protibia **F** Mesotrochanter and mesofemur **G** Metatrochanter and metafemur **H** Sternite IX **I–K** Aedeagus, in dorsal view (**I**), lateral (**J**), and ventral (**K**) view. Scale bars: 0.3 mm (**A, B, D, F, G**); 0.2 mm (**C, I, J, K**); 0.05 mm (**E**); 0.1 mm (**H**).

Male (Figure 1B). Length 2.61mm. Head longer than wide, HL 0.57 mm, HW 0.51 mm; eyes each composed of about 18 facets. Antenna with scape about 2.8 times as long as wide, antennomeres II–VIII similar, each about as long as wide, antennomeres IX–XI enlarged, simple (Figure 3A). Pronotum (Figure 3B) about as long as wide, PL 0.53 mm, PW 0.52 mm. Elytra much wider than long, EL 0.64 mm, EW 0.90 mm. Metaventral processes (Figure 3C) short, pointed apically. Protrochanter and profemur (Figure 3D) simple, protibia with large, triangular spine at apex (Figure 3E); mesotrochanter and mesofemur simple (Figure 3F); metatrochanter (Figure 3G) with blunt, apically curved ventral projection. Abdomen slightly wider than elytra, AL 0.93 mm, AW 1.00 mm; tergite IV about twice as long as tergite V; sternite IX as in Figure 3H. Length of aedeagus (Figure 3I–J) 0.48 mm; median lobe asymmetric, narrowing apically; elongate parameres expanded dorso-ventrally, slightly curved ventrally at basal 2/5 in lateral view.

Female. Similar to male in general morphology; eyes each composed of about 18 facets; antennae and legs simple; lacking metaventral processes. Measurements: HL 0.57 mm, HW 0.51 mm, PL 0.52 mm, PW 0.51 mm, EL 0.58 mm, EW 0.94 mm, AL 0.93 mm, AW 1.00 mm.

#### Distribution.

China: Guizhou (Figure 11B).

#### Etymology.

The specific epithet refers to the large apical spine of the protibia.

#### Comparative notes.

*Linandenticulatus* is placed as a member of the *L.chinensis*-group based on the unmodified male antennomeres IX–X, and externally resembles *L.hujiayaoi* Yin & Li from Guangxi. These two species share a similar form of the antennal club, short metaventral processes, and the blunt, apically curved ventral projection of the metatrochanter. They can be best separated by the much more distinct apical projection of the protibia, and median lobe of the aedeagus with a strongly narrowed apical part and much broader parameres in the new species.

### Linan divaricatus sp. n.

Taxon classificationAnimaliaColeopteraStaphylinidae

http://zoobank.org/8D5A2E97-1E58-4A47-AFEC-268427873AC

Figs 4A , 5 , 11A

#### Type material.

(7 ♂♂). **Holotype: CHINA**: ♂: ‘China: W. Jiangxi Province, Luxi County (芦溪县), Wugong Shan (武功山), 27°27'53"N, 114°10'47"E, mixed forest, leaf litter, wood sifted & beating, ca. 1570 m, 27.x.2013, Peng, Shen & Yan leg.’(SNUC). **Paratype: CHINA**: 3 ♂♂, same label data as holotype; 1 ♂, Jiangxi, Pingxiang City, Wugong Shan National Park, 27°27'55"N, 114°09'58"E, cableway station to Baoshui Waterfall, broad leaf, sifted, 1000–1350 m, 20.vii.2013, Song, Yin, Yu leg.’; 2 ♂♂, ‘China: W. Jiangxi Province, Luxi County, Yangshimu(羊狮幕), 27°33'38"N, 114°14'35"E, mixed forest, leaf litter, wood sifted & beating, ca. 1580m, 25.x.2013, Peng, Shen & Yan leg.’ (all in SNUC).

#### Diagnosis of male.

Length 2.74–2.82 mm; antennomeres IX–X strongly modified, antennomere IX angulate at anterolateral corner, obliquely connecting with strongly transverse antennomere X; broad metaventral processes bifurcate at apex; protibia with small apical spine; mesotrochanter with tiny ventral spine.

#### Description.

**Figure 4.**
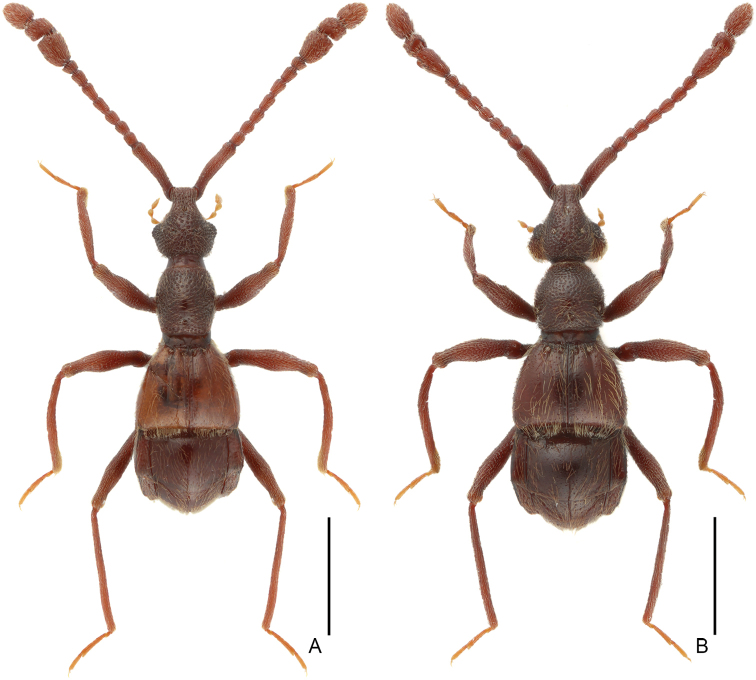
Dorsal habitus of *Linan* species **A***L.divaricatus* sp. n. **B***L.geneolatus* sp. n. Scale bars: 1 mm.

**Figure 5.**
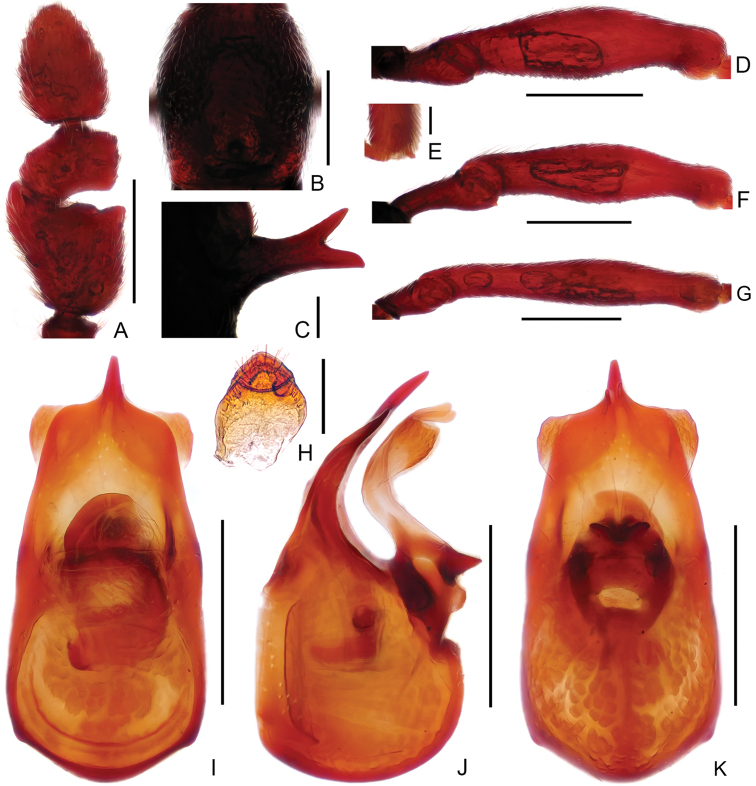
Diagnostic features of male *Linandivaricatus* sp. n. **A** Antennal club **B** Pronotum **C** Metaventral process, lateral view **D** Protrochanter and profemur **E** Apex of protibia **F** Mesotrochanter and mesofemur **G** Metatrochanter and metafemur **H** Sternite IX **I–K** Aedeagus, in dorsal view (**I**), lateral (**J**), and ventral (**K**) view. Scale bars: 0.3 mm (**A, B, D, F, G**); 0.1 mm (**C, H**); 0.05 mm (**E**); 0.2 mm (**I, J, K**).

Male (Figure 4A). Length 2.74–2.82 mm. Head longer than wide, HL 0.60–0.64 mm, HW 0.55–0.56 mm; eyes each composed of about 30 facets. Antenna with scape about 4.2 times as long as wide, antennomeres II–III and VIII similar, each about as long as wide, IV–VII each longer than wide, antennomere IX (Figure 5A) strongly expanded, angulate at anterolateral corner, antennomere X strongly transverse, obliquely connecting with IX. Pronotum (Figure 5B) slightly longer than wide, PL 0.56–0.57 mm, PW 0.49–0.51 mm. Elytra much wider than long, EL 0.62–0.64 mm, EW 0.87–0.90 mm. Metaventral processes (Figure 5C) broad, bifurcate at apex in lateral view. Protrochanter and profemur simple (Figure 5D), protibia with indistinct spine (Figure 5E) at apex; mesotrochanter (Figure 5F) with tiny ventral spine, mesofemur simple; metatrochanter and metafemur simple (Figure 5G). Abdomen slightly wider than elytra, AL 0.96–0.97mm, AW 0.89–0.93 mm; tergite IV about twice as long as tergite V; sternite IX as in Figure 5H. Length of aedeagus (Figure 5I–K) 0.45 mm; median lobe symmetric, pointed apically at middle; parameres strongly curved ventrally and constricted at apices in lateral view.

Female. Unknown.

#### Distribution.

China: Jiangxi (Figure 11A).

#### Etymology.

The new specific epithet refers to the long and bifurcate metaventral processes.

#### Comparative notes.

The new species belongs to the *L.cardialis*-group based on the strongly modified male antennomere IX. *Linandivaricatus* is most similar to *L.huapingensis* and *L.geneolatus* sp. n. (described below) in sharing both the angulate anterolateral corner of male antennomere IX, and median lobe of aedeagus strongly constricted at middle of the apex. From both latter species the new species differs by the relatively much broader antennomere IX, much broader metaventral processes bifurcate at the apex, and simple metatrochanter. In *L.geneolatus* sp. n., the male antennomere X is also strongly excavate at basal half.

### Linan geneolatus sp. n.

Taxon classificationAnimaliaColeopteraStaphylinidae

http://zoobank.org/1BC5BDEC-1AAB-4B39-94C6-0F973EE70C5A

Figs 4B , 6 , 11A

#### Type material.

(1 ♂). **Holotype: CHINA**: ♂: ‘China: N. Guizhou, Daozhen County, Dashahe N. R., 29°10'12"N, 107°33'36"E, mixed leaf litter, sifted, 1730 m, 07.VII.2015, Jiang, Peng, Tu, & Zhou leg.’ (SNUC).

#### Diagnosis of male.

Length 3.06 mm; postgenae broadly expanded laterally; antennomeres IX–X strongly modified, IX strongly projecting at anterolateral corner, X broadly concave at basal half; metaventral processes short; metatrochanter with short, blunt ventral projection.

#### Description.

**Figure 6.**
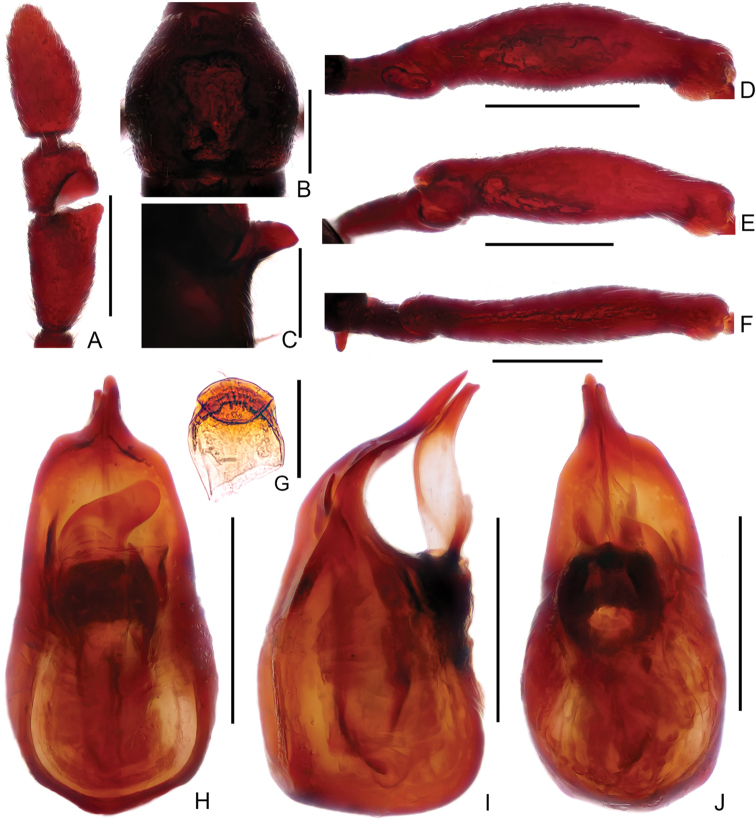
Diagnostic features of male *Linangeneolatus* sp. n. **A** Antennal club **B** Pronotum **C** Metaventral process, lateral view **D** Protrochanter and profemur **E** Mesotrochanter and mesofemur **F** Metatrochanter and metafemur **G** Sternite IX **H–J** Aedeagus, in dorsal view (**H**), lateral (**I**), ventral (**J**) view. Scale bars: 0.3 mm (**A, B, D, E, F**); 0.2 mm (**C, H, I, J**); 0.1 mm (**G**).

Male (Figure 4B). Length 3.06 mm. Head slightly longer than wide, HL 0.69 mm, HW 0.66 mm; postgenae expanded laterally; eyes prominent, each composed of about 22 facets. Antenna with scape about 3.8 times as long as wide, antennomeres II–IV and VIII similar, each about as long as wide, V–VII each slightly longer than wide, antennomere IX (Figure 6A) broad, strongly projecting at anterolateral corner, antennomere X transverse, broadly concave at basal half. Pronotum (Figure 6B) about as long as wide, PL 0.60 mm, PW 0.59 mm. Elytra much wider than long, EL 0.69 mm, EW 0.98 mm. Metaventral processes (Figure 6C) short, narrowing at apex in lateral view. Protrochanter and profemur (Figure 6D), and mesotrochanter and mesofemur (Figure 6E) simple; metatrochanter (Figure 6F) with short, blunt ventral projection. Abdomen slightly wider than elytra, AL 0.99 mm, AW 1.06 mm; tergite IV about twice as long as tergite V; sternite IX as in Figure 6G. Length of aedeagus (Figure 6H–J) 0.44 mm; median lobe nearly symmetric, strongly constricted at middle of apex.

Female. Unknown.

#### Distribution.

China: Guizhou (Figure 11A).

#### Etymology.

The new specific epithet refers to the strongly expanded postocular margins.

#### Comparative notes.

The new species is placed as a member of the *L.cardialis*-group based on the strongly modified antennomere IX in the male, and is most similar to *L.huapingensis* in the shape of antennomere IX and spinose metatrochanter in the male. These two species can be separated by antennomere X being strongly excavate at the basal half, and the short metaventral processes in males of the new species, while in *L.huapingensis* the antennomere X lacks an excavation, and the metaventral processes are much longer and thinner. Otherwise, *Linangeneolatus* is the only member of the genus that exhibits broadened postgenae, which makes it readily separable from all other congeners.

### Linan mangshanus sp. n.

Taxon classificationAnimaliaColeopteraStaphylinidae

http://zoobank.org/05B40319-57BA-4391-8A67-17E88B068130

Figs 7A , 8 , 11B

#### Type material.

(2 ♂♂). **Holotype: CHINA**: ♂: ‘China: Hunan, Chenzhou, Yizhang Hsien (宜章县), Mangshan N. R. (莽山自然保护区), 24°56'26"N, 112°59'18"E, mixed forest, leaf litter, wood sifted & beating, 1400 m, 26.iv.2015, Peng, Tu, Zhou leg.’ (SNUC). **Paratype: CHINA**: 1 ♂, same label data as the holotype, (SNUC).

#### Diagnosis of male.

Length 3.25–3.35 mm; antennomere IX–X strongly modified, IX strongly expanded and bent at lateral margin, with distinct process at anteromesal corner; broad metaventral processes expanded at apex in lateral view; metacoxa with roundly triangular ventral projection.

#### Redescription.

**Figure 7.**
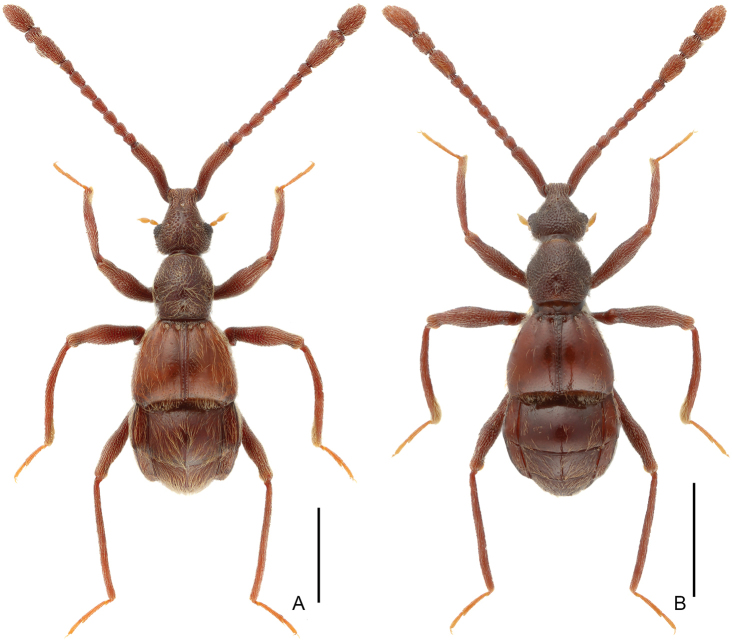
Dorsal habitus of *Linan* species. **A***L.mangshanus* sp. n. **B***L.mulunensis* sp. n. Scale bars: 1 mm.

**Figure 8.**
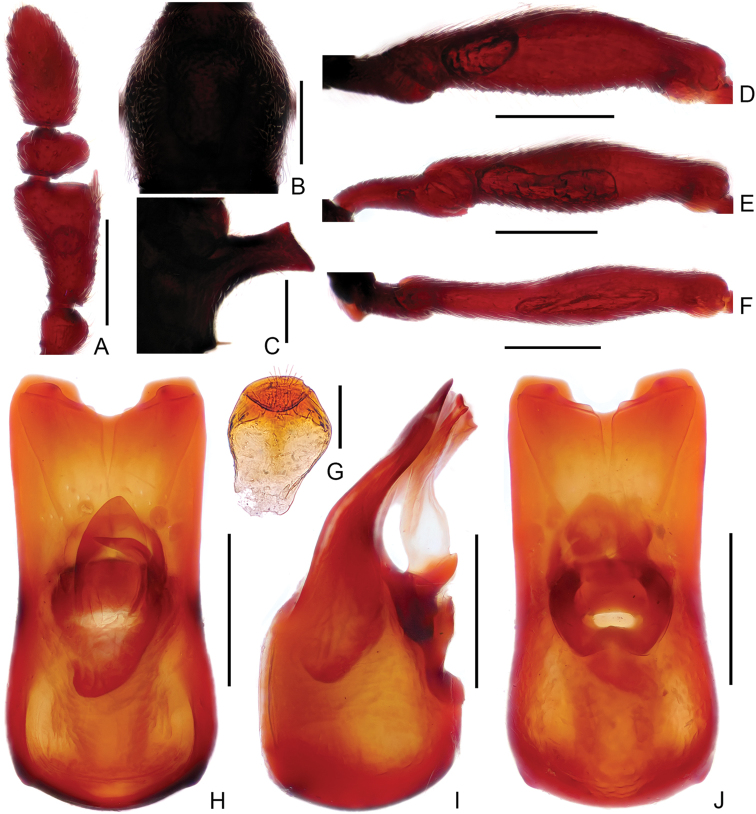
Diagnostic features of male *Linanmangshanus* sp. n. **A** Antennal club **B** Pronotum **C** Metaventral process, lateral view **D** Protrochanter and profemur **E** Mesotrochanter and mesofemur **F** Metatrochanter and metafemur **G** Sternite IX **H–J.** Aedeagus, in dorsal view (**H**), lateral (**I**), ventral (**J**) view. Scale bars: 0.3 mm (**A, B, D, E, F**); 0.2 mm (**C, H, I, J**); 0.1 mm (**G**).

Male (Figure 7A). Length 3.25–3.35 mm. Head longer than wide, HL 0.69–0.75 mm, HW 0.60–0.64 mm; eyes prominent, each composed of about 33 facets. Antenna with scape about 3.8 times as long as wide, antennomeres II–III and VIII similar, each about as long as wide, IV slightly longer than wide, V–VII each much longer than wide, antennomere IX (Figure 8A) much longer than wide, strongly expanded and bent at lateral margin, with distinct process at anteromesal corner, antennomere X strongly transverse. Pronotum (Figure 8B) slightly longer than wide, PL 0.65–0.66 mm, PW 0.60–0.63 mm. Elytra much wider than long, EL 0.71–0.73 mm, EW 1.07–1.08 mm. Metaventral processes (Figure 8C) broad, expanded at apex in lateral view. Protrochanter, profemur simple (Figure 8D), mesotrochanter, and mesofemur (Figure 8E) simple; metacoxa (Figure 8F) with short and triangular ventral projection. Abdomen slightly wider than elytra, AL 1.20–1.21mm, AW 1.12–1.13 mm; tergite IV about twice as long as tergite V; sternite IX as in Figure 8G. Length of aedeagus (Figure 8H–J) 0.56 mm; median lobe symmetric; elongate parameres slightly curved ventrally at middle in lateral view.

Female. Unknown.

#### Distribution.

China: Hunan (Figure 11B).

#### Etymology.

The new species is named after the type locality, i.e., Mangshan Nature Reserve.

#### Comparative notes.

The new species is placed as a member of the *L.cardialis*-group based on the modified male antennomere IX, and most similar to *L.hainanicus* Hlaváč in shape of male antennomere IX. They can be readily separated by the much broader metaventral processes, lack of a large apical projection of the protibia, complete symmetric aedeagal median lobe, and different structures of the endophallus of the new species. The broad metaventral processes, projecting metacoxae, and high symmetry of the aedeagal median lobe are shared by *L.uenoi* Yin & Nomura from Guangxi. They differ mainly by the modified antennomeres VII–VIII of *L.uenoi*, a quite distinct feature for a member of *Linan*.

### Linan mulunensis sp. n.

Taxon classificationAnimaliaColeopteraStaphylinidae

http://zoobank.org/2129E81A-E3FD-479E-82D8-E9313AEF52D3

Figs 7B , 9 , 11B

#### Type material.

(2 ♂♂). **Holotype: CHINA**: ♂: ‘China: Guangxi, Hechi City, Mulun N. R. (木论自然保护区), 25°12'14"N, 108°5'46"E, mixed leaf litter, sifted, 460 m, 27.VII.2015, Chen, He, & Hu leg.’ (SNUC). **Paratype: China**: 1 ♂, same label data as holotype, (SNUC).

#### Diagnosis of male.

Length 2.75–2.77 mm; antennomeres IX–XI elongate, lacking modification; long metaventral processes narrowed apically; protibia with acute apical spine; metacoxa with large, apically narrowing and blunt ventral projection.

#### Description.

**Figure 9.**
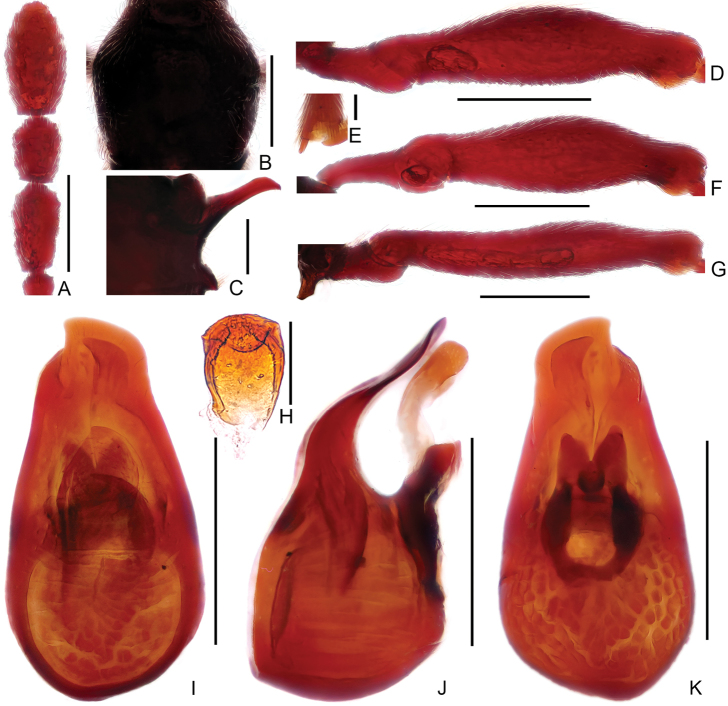
Diagnostic features of male *Linanmulunensis* sp. n. **A** Antennal club **B** Pronotum **C** Metaventral process, lateral view **D** Protrochanter and profemur **E** Apical spur of protibia **F** Mesotrochanter and mesofemur **G** Metatrochanter and metafemur **H** Sternite IX **I–K** Aedeagus, in dorsal view (**I**), lateral (**J**), ventral (**K**) view. Scale bars: 0.3 mm (**A, B, D, F, G**); 0.2 mm (**C, I, J, K**); 0.05 mm (**E**); 0.1 mm (**H**).

Male (Figure 7B). Length 2.75–2.78 mm. Head slightly longer than wide, HL 0.59–0.60 mm, HW 0.52–0.53 mm; eyes small, each composed of about 18 facets. Antennal scape about 4.0 times as long as wide, antennomeres V–VII slightly longer than II–IV and VIII, antennomeres IX–XI simple (Figure 9A). Pronotum (Figure 9B) about as long as wide, PL 0.55–0.56 mm, PW 0.55–0.56 mm. Elytra much wider than long, EL 0.66–0.70 mm, EW 0.93–0.95 mm. Metaventral processes (Figure 9C) long, narrowed apically, with short, distinct protuberance above metacoxae. Protrochanter and profemur simple (Figure 9D); protibia with small but distinct spine (Figure 9E) at apex; mesotrochanter and mesofemur simple (Figure 9F); metacoxa (Figure 9G) with blunt, apically narrowed ventral projection. Abdomen slightly wider than elytra, AL 0.93–0.94 mm, AW 0.97–0.98 mm; tergite IV about twice as long as tergite V; sternite IX as in Figure 9H. Length of aedeagus (Figure 9I–K) 0.37 mm; median lobe asymmetric at apex, narrowing apically; parameres strongly curved in lateral view.

Female. Unknown.

#### Distribution.

China: Guangxi Province (Figure 11B).

#### Etymology.

The new species is named after the type locality, i.e., Mulun Nature Reserve.

#### Comparative notes.

The new species is placed as a member of the *L.chinensis*-group based on the simple male antennomeres IX–X. The form and proportions of antennomeres IX–XI are similar to *L.chinensis* (Löbl) and *L.inornatus* Yin & Li. However, both known species lack a projection on the ventral margin of metacoxa in the males, where there is a large, apically narrowed projection for the new species.

### Linan megalobus

Taxon classificationAnimaliaColeopteraStaphylinidae

Yin & Li, 2011 in Yin et al. 2011

Figs 10 , 11B

Linan megalobus Yin & Li, 2011 in Yin et al. 2011: 132.

#### Additional material examined.

1 ♂, 1 ♀, ‘China: Hubei, Enshi Tujia and Miao Autonomous Prefecture, Xingdoushan N. R., San-xian-chang, 30°2'20.48"N, 109°8'33.89"E, 1114 m, 20.v.2017, sift, Zhou GC, Tian T, & Huang ZG leg.’ (SNUC).

#### Distribution.

China: Guizhou, Hubei (**new provincial record**) (Figure 11B).

#### Discussion.

**Figure 10.**
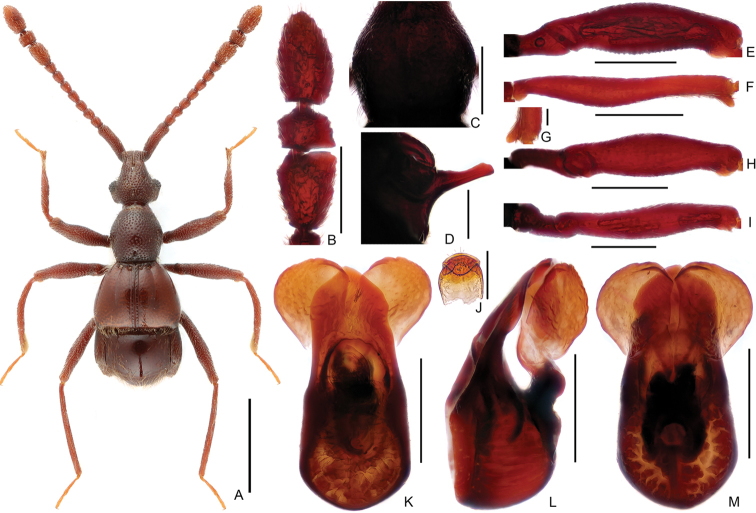
Diagnostic features of male *Linanmegalobus*. **A** Dorsal habitus **B** Antennal club **C** Pronotum **D** Metaventral process, lateral view **E** Protrochanter and profemur **F** Protibia **G** Apical spur of protibia **H** Mesotrochanter and mesofemur **I** Metatrochanter and metafemur **J** Sternite IX **K–M** Aedeagus, in dorsal view (**K**), lateral (**L**), ventral (**M**) view. Scale bars: 1 mm (**A**); 0.3 mm (**B, C, E, F, H, I**); 0.2 mm (**D, K, L, M**); 0.05 mm (**G**); 0.1 mm (**J**).

*Linanmegalobus* was originally described from Kuankuoshui Nature Reserve (宽阔水自然保护区) in Guizhou, and placed as a member of the *L.cardialis*-group (Yin et al. 2011). The population from Hubei shows little variation in external morphology (Figure 10A–J) compared to that from the type locality, but possesses distinctly broader parameres of the aedeagus (Figure 10K–M). The present new record extends the range of this species some 280 km to the southwest.

**Figure 11.**
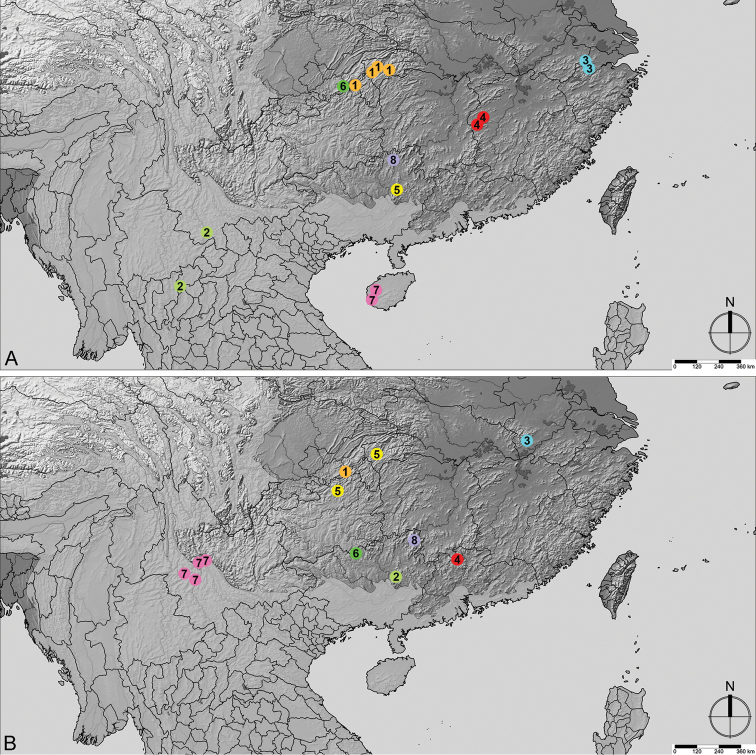
Distribution of *Linan* species. **A***L.arcitibialis* (1), *L.cardialis* (2), *L.chinensis* (3), *L.divaricatus* (4), *L.fortunatus* (5), *L.geneolatus* (6), *L.hainanicus* (7), *L.huapingensis* (8) **B***L.denticulatus* (1), *L.hujiayaoi* (2), *L.inornatus* (3), *L.mangshanus* (4), *L.megalobus* (5), *L.mulunensis* (6), *L.tendothorax* (7), *L.uenoi* (8).

##### Key to males

**Table:** 

1	Antennae with antennomeres IX or X strongly modified	**2 (*L.cardialis*-group)**
–	Antennae with antennomeres IX–X simple, not modified	**12 (*L.chinensis*-group)**
2	Antennomere IX angularly expanded laterally at basal third, with small rounded process near apex (Fig. 2A); mesotibiae strongly arched (Fig. 2G). (China: Hubei, Chongqing; Fig. 11A)	***L.arcitibialis* sp. n.**
–	Antennomere IX strongly broadened apically, never expanded at basal third, often angularly projecting at apex; mesotibiae only slightly arched	**3**
3	Median lobe of aedeagus strongly and abruptly constricted at middle of apex	**4**
–	Median lobe of aedeagus laterally bent, or emarginate at middle of apex	**6**
4	Antennomere X broadly concave at basal half (Fig. 6A); postgenae broadly expanded laterally (Fig. 4B). (China: Guizhou; Fig. 11A)	***L.geneolatus* sp. n.**
–	Antennomere X lacking excavation; postgenae roundly constricted posteriorly	**5**
5	Antennomere VIII slightly transverse (Fig. 4A), IX about as long as wide (Fig. 5A); metaventral processes thick, bifurcate at apex in lateral view (Fig. 5C); protibia with indistinct apical spine (Fig. 5E); metatrochanter simple (Fig. 5G). (China: Jiangxi; Fig. 11A)	***L.divaricatus* sp. n.**
–	Antennomere VIII about as long as wide, IX much longer than wide (Yin and Li 2013: 149, fig. 6A); metaventral processes thin, rounded at apex in lateral view (Yin and Li 2013: 149, fig. 6C); protibia simple; metatrochanter with short triangular ventral spine (Yin and Li 2013: 149, fig. 6F). (China: Guangxi; Fig. 11A)	***L.huapingensis* Yin & Li**
6	Median lobe of aedeagus nearly symmetric, broadly emarginate at middle of apex	**7**
–	Median lobe of aedeagus asymmetric, usually narrowing and bent to right (morphological position) at apex, never emarginate at middle	**8**
7	Antennomeres VII–VIII strongly modified, IX about 1.45 times as long as wide, slightly angulate at anterolateral corner (Yin et al. 2013: 351, fig. 29); protibia with small apical spine (Yin et al. 2013: 351, fig. 33); aedeagus (Yin et al. 2013: 351, figs 37–39) relatively stouter, length / width about 1.75. (China: Guangxi; Fig. 11B)	***L.uenoi* Yin & Nomura**
–	Antennomeres VII–VIII simple, IX about 1.20 times as long as wide, with long acute projection at anterolateral corner (Fig. 8A); protibia simple; aedeagus (Fig. 8H–J) relatively more slender, length / width about 2.25. (China: Hunan; Fig. 11B)	***L.mangshanus* sp. n.**
8	Pronotal lateral margins roundly expanded basolaterally (Yin and Li 2012: 94, fig. 6D). (China: Yunnan; Fig. 11B)	***L.tendothorax* Yin & Li**
–	Pronotal lateral margins evenly rounded laterally, not expanded basolaterally	**9**
9	Pronotal and elytral basolateral margins densely setose (Yin and Li 2013: 146, fig. 4A). (China: Guangxi; Fig. 11A)	***L.fortunatus* Yin & Li**
–	Pronotal and elytral basolateral margins lacking dense setae	**10**
10	Antennomere IX strongly bent at lateral margin (Yin et al. 2011: 128, fig. 9). (China: Hainan; Fig. 11A)	***L.hainanicus* Hlaváč**
–	Antennomere IX straight or slightly broadened at lateral margin	**11**
11	Pro- and mesotrochanter with distinct, pointed ventral spine (Yin et al. 2011: 130, figs 22, 23); protibia with short, bluntly rounded protuberance at apex; aedeagus with short and narrow parameres (Yin et al. 2011: 131, figs 27, 28). (China: Yunnan; Thailand: Wiang Pa Pao; Fig. 11A)	***L.cardialis* Hlaváč**
–	Pro- and mesotrochanter simple; protibia with elongate, rounded protuberance at apex (Yin et al. 2011: 131, fig. 26); aedeagus with long, apically strongly broadened parameres (Fig. 10K–M, Yin et al. 2011: 131, figs 35–36). (China: Hubei; Guizhou, Fig. 11B)	***L.megalobus* Yin & Li**
12	Antennomere IX slightly to moderately transverse	**13**
–	Antennomere IX slightly to distinctly elongate	**14**
13	Antennomere VIII about as long as wide (Fig. 3A); protibia with distinct apical spine (Fig. 3E); median lobe of aedeagus asymmetric, strongly narrowed at apical fourth (Fig. 3I–K). (China: Guizhou; Fig. 11B)	***L.denticulatus* sp. n.**
–	Antennomere VIII moderately transverse (Yin and Li 2013: 150, fig. 7A); protibia simple, lacking spine at apex; median lobe of aedeagus nearly symmetric, uniformly narrowing from middle toward apex (Yin and Li 2013: 150, fig. 7H–J). (China: Guangxi; Fig. 11B)	***L.hujiayaoi* Yin & Li**
14	Metaventrite with short, distinct protuberances above metacoxae (Fig. 9C); protibia with small, acute apical spine (Fig. 9E); metacoxa with large, apically narrowing and blunt ventral projection (Fig. 9G). (China: Guangxi; Fig. 11B)	***L.mulunensis* sp. n.**
–	Metaventrite lacking protuberances above metacoxae; protibia lacking apical spine; metacoxa simple	**15**
15	Metaventral processes relatively shorter, narrowing at apex (Yin et al. 2011: 128, fig. 18); elytra and abdomen relatively broader in contrast to pronotum (PW : EW : AW = 1.00 : 1.88–1.90 : 2.02–2.03) (Yin et al. 2011: 128, fig. 2). (China: Zhejiang; Fig. 11A)	***L.chinensis* (Löbl)**
–	Metaventral processes relatively much longer, broad at apex (Yin et al. 2011: 128, fig. 20); elytra and abdomen relatively narrower in contrast to pronotum (PW : EW : AW = 1.00 : 1.62–1.64 : 1.75–1.78) (Yin et al. 2011: 128, fig. 4). (China: Anhui; Fig. 11B)	***L.inornatus* Yin & Li**

## Supplementary Material

XML Treatment for Linan arcitibialis

XML Treatment for Linan denticulatus

XML Treatment for Linan divaricatus

XML Treatment for Linan geneolatus

XML Treatment for Linan mangshanus

XML Treatment for Linan mulunensis

XML Treatment for Linan megalobus
